# The cockroach genus *Sorineuchora* Caudell, 1927 from China (Blattodea, Ectobiidae, Pseudophyllodromiinae)

**DOI:** 10.3897/zookeys.697.13617

**Published:** 2017-09-15

**Authors:** Meng Li, Yan-Li Che, Yu-Hong Zheng, Zong-Qing Wang

**Affiliations:** 1 Institute of Entomology, College of Plant Protection, Southwest University, Beibei, Chongqing 400716, China

**Keywords:** Blattellidae, distribution, key, new species, *Sorineuchora*

## Abstract

In this paper, three new species (*S. bimaculata* **sp. n.**, *S. viridis* **sp. n.**, and *S. hispida* **sp. n.**) and five known species, *S. formosana* (Matsumura, 1913), *S. nigra* (Shiraki, 1908), *S. shanensis* (Princis, 1950), *S. bivitta* (Bey-Bienko, 1969), and *S. undulata* (Bey-Bienko, 1958), are described and illustrated. *Sorineuchora undulata* was previously synonymized with *S. nigra*, and is now reinstated as a valid species. A key to the males of *Sorineuchora* from China is provided.

## Introduction

The cockroach genus *Sorineuchora* was established by Caudell (1927), and synonymized with *Chorisoneura* Brunner von Wattenwyl, 1865 by Hebard (1929). However, comparing *Sorineuchora* with *Chorisoneura*, Brujning (1948) pointed out there are obvious differences in the hind-wing venation and apical triangle. Subsequently, Asahina (1978) discussed the interspecific relationships of *Sorineuchora* and considered *S. formosana* (Matsumura, 1913) and *S. setshuana* (Bey-Bienko, 1958) to be closely related to *S. lativitrea* (Walker, 1868) and *S. nigra* (Shiraki, 1908), respectively. At the same time, the seven other known species were not treated. Later, Roth (1998) revised *Sorineuchora* and recorded eleven species worldwide, of which nine species were from China including the four species mentioned above in Asahina (1978). Liu and Zhu (2001), who recorded *Sorineuchora* species under *Chorisoneura*, synonymized *S. setshuana* and *S. undulata* with *S. nigra* without giving any details. Recently, Che et al. (2017) showed that the subfamily Pseudophyllodromiinae was a polyphyletic group, and *S. nigra* and *S. bivitta* (Bey-Bienko, 1969) formed monophyletic groups. Wang et al. (2017) indicated that *Balta* and *Sorineuchora* are more closely related to each other than either is to *Allacta*, *Shelfordina*, or *Latiblattella*.

Recently, specimens deposited in Southwest University and Hebei University were examined, and eight species of *Sorineuchora* identified from China including five known and three new species. Because of the lack of specimens and male description of *S. punctipennis* (Princis, 1950), the species is not included in the key, only recorded as information under the remarks of *S. undulata*. These cockroaches were mostly attracted by light at night (Fig. 10A–B), but were also found on vegetation such as leaves (Fig. 10C) and flowers (Fig. 10D).

## Materials and methods

Male genital segments were macerated in 10% NaOH for one hour, and rinsed with distilled water, then stored in glycerine for dissection and observation. Line drawings were made with a Motic K400 stereomicroscope. Habitus photos were taken with a Canon 50D plus a Canon EF100mm f/2.8L Macro IS USM lens, and stacked with Helicon Focus software. The map was made with Natural Earth (http://www.naturalearthdata.com). All photos and images were edited with Adobe Photoshop CS6.

COI sequence (KY349518 and KY349519) of *S. nigra* was downloaded from GenBank to compare with COI sequence of the exceptional female specimen (Fig. 10C) (Accession number: MF612149).

Morphological terminology mainly follows Roth (2003), and wing venation and genitalia terms are according to Li and Wang (2015) and McKittrick (1964), respectively. The vein abbreviations in this article are listed as below following Li and Wang (2015):

**CuA** cubitus anterior

**M** media

**R** radius

**RA** radius anterior

**RP** radius posterior

**Sc** subcosta

Specimens examined are deposited in the following collections. **IESWU** Institute of Entomology, Southwest University (西南大学昆虫研究所), Beibei, Chongqing, China; **MHBU** Museum of Hebei University (河北大学博物馆), Baoding, Hebei, China.

## Taxonomy

### Genus Sorineuchora

Taxon classificationAnimaliaBlattodeaEctobiidae

Caudell, 1927

#### Type species.

*Sorineuchora javanica* Caudell, 1927.

#### Diagnosis.

(Partly after Roth (1998)). Fifth segment of maxillary palpus longer than the fourth. Pronotum subelliptical. Front femur with a row of small piliform spinules and two large distal spines (Type C_2_); proximal four tarsomeres with tarsal pulvilli, tarsal claws simple, asymmetrical, of different size. Tegmina and wings fully developed extending beyond end of abdomen, hind-wing R with oblique branches, M distinct, CuA with one to three branches, apical triangle or appendicular field present, sometimes subobsolete (*S. javanica* and *S. viridis* sp. n.). Abdominal terga of male unspecialized. Supra-anal plate symmetrical, hind margin convexly rounded; paraprocts simple, sheet-like. Subgenital plate with subsymmetrical hind margin. Phallomere L1 consisting of several irregular sclerites. Genital hook on the right side (the diagnosis of subfamily).

According to Roth (1998) there is a close relationship among *Chorisoneura* Brunner von Wattenwyl, 1865, *Chorisoneurodes* Princis, 1962, *Chorisoserrata* Roth, 1998 and *Sorineuchora* Caudell, 1927. Roth (1998) differentiated *Sorineuchora* from *Chorisoneura* and *Chorisoneurodes* by the unspecialized terga in *Sorineuchora*. *Sorineuchora* also has the following traits that differentiate it from *Chorisoserrata*: asymmetrical tarsal claws; interocular vertex not truncate, the fourth maxillary palpomere not longer than the fifth; and antero-ventral margin of forefemur with two apical spines.

Many similar morphological traits exist among *Balta* Tepper, 1893 and *Sorineuchora*, such as proximal four tarsomeres with tarsal pulvilli, tarsal claws asymmetrical and unspecialized, and abdominal terga of male unspecialized. According to the maximum likelihood COI tree in Che et al. (2017) and the combined data (12SrRNA, 16SrRNA, COII, 28SrRNA and H3) tree in Wang et al. (2017), there is a close relationship between *Sorineuchora* and *Balta*. *Sorineuchora* can be distinguished from *Balta* by the following characters: bodies of the former are generally less wide in dorsal view, in the former the fourth maxillary palpomere is not longer than the fifth, and smaller V shaped incision of the hind margin of the subgenital plate. Further study is needed to distinguish the two.

#### Remarks.

Species of *Sorineuchora* have strikingly variable morphology. The body coloration ranges from pale green to black (Figs 10, 11, 12); the markings on the pronotal disk vary greatly; the shape of their styli is either cylindrical (Figs 2F, 4E, 6H, 8E) or conical (Figs 3E, 5G, 7H, 9E); the shape of sclerites of L2vm is highly variable, some are filamentary, and some are rod-like. Given this variation, the genus *Sorineuchora* might be not monophyletic, revision based on characters of the type specimen of the genus or molecular data is needed.

#### Distribution.

Oriental and Palaearctic regions.

#### Key to the males of *Sorineuchora* from China

**Table:** 

1	Body light-colored, yellow or pale green (Figs 10A, 11A–D, 12E–F)	**2**
–	Body color comparatively dark (Figs 10B–D, 11F–K, 12A–D, 12G–J)	**3**
2	Uniformly yellowish white (dried specimen) (Fig. 12E–F) or light green (alive) (Fig. 10A)	***S. viridis* sp. n.**
–	Body yellowish brown or straw-yellow	**8**
3	Pronotal disk black, with white or yellow symmetrical stripes, and vertex dark with a pair of white or yellow stripes (Figs 4B, 10D, 11H)	***S. shanensis***
–	Pronotal disk without stripes, or some with stripes unlike those above	**4**
4	Tegmina yellowish brown with four dark spots on the radius and many black dots on veins (Figs 5D, 11J)	***S. undulata***
–	Tegmina without spots like those above	**5**
5	Vertex with two round yellowish brown spots on the middle (Fig. 7C)	***S. bimaculata* sp. n.**
–	Vertex without spots or with spots unlike those above	**6**
6	Vertex with a white stripe or a rudimentary dark stripe or without stripes	**7**
–	Vertex with two black stripes, the regions between them yellow (Figs 6B, 12B)	***S. bivitta***
7	Pronotal disk dark with a rudimentary dark stripe or without stripes	***S. nigra***
–	Pronotal disk brown, with a yellowish brown, longitudinal stripe (Figs 9B, 12G)	***S. hispida* sp. n.**
8	Subgenital plate with an incision slightly to the left of the middle. Left stylus bent out toward the left apically and pointed and is longer than the right stylus	***S. pallens*^1^**
–	Subgenital plate with an incision medially. Left and right styli are similar and cylindrical (Fig. 2F)	**9**
9	L2vm apex with one branch, and R3 lying under the L2vm (Fig. 2H)	***S. formosana***
–	L2vm without branch and R3 (Roth, 1998, fig. 37)	***S. lativitrea***
	**^1^**From Bey-Bienko (1969). *Sorineuchora pallens* is not described in the current paper because no specimens were examined.

### Sorineuchora formosana

Taxon classificationAnimaliaBlattodeaEctobiidae

(Matsumura, 1913)

Figs 2 , 11A–B

Chorisoneura formosana Matsumura, 1913: 14, pl. 2, fig. 13 (♀); Asahina 1978: 235 (♂♀).
Theganopteryx formosana (Matsumura): Shiraki 1931: 209 (♂♀).
Sorineuchora formosana (Matsumura): Roth 1998: 15 (♂♀).

#### Material examined

**(all deposited in IESWU). Yunnan**: 1 male, Xishuangbanna, Tropical Botanical Garden, 593 m, 12 November 2009, Guo Tang leg.; 1 male, Mengzi, Lvshuihe first hydroelectric station, 470 m, 19 April 2009, Wei-Wei Zhang leg.; 1 male, Simao, 01 May 2012, Li-Chao Tian leg.; 1 female 1 male, Xishuangbanna, National Nature Reserve, 736 m, 18 August 2012, Guo Zheng leg.; 1 male, Xishuangbanna, Mengla, 1200–1400 m, 10 May 1958, Chun-Pei Hong leg. **Hainan**: 1 male, Tongzha, 07 June 1963, Ya-Lin Zhang leg.; 1 male, Ledong, Mt. Jianfengling, 1050 m, 06–09 December 2007, Wei-Wei Zhang leg.

**Figure 1.**
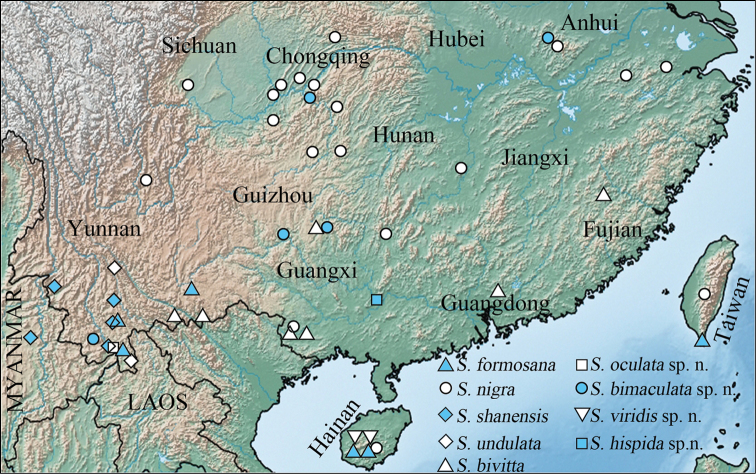
Known occurrences of *Sorineuchora* in China and Myanmar.

#### Diagnosis.

**Figure 2.**
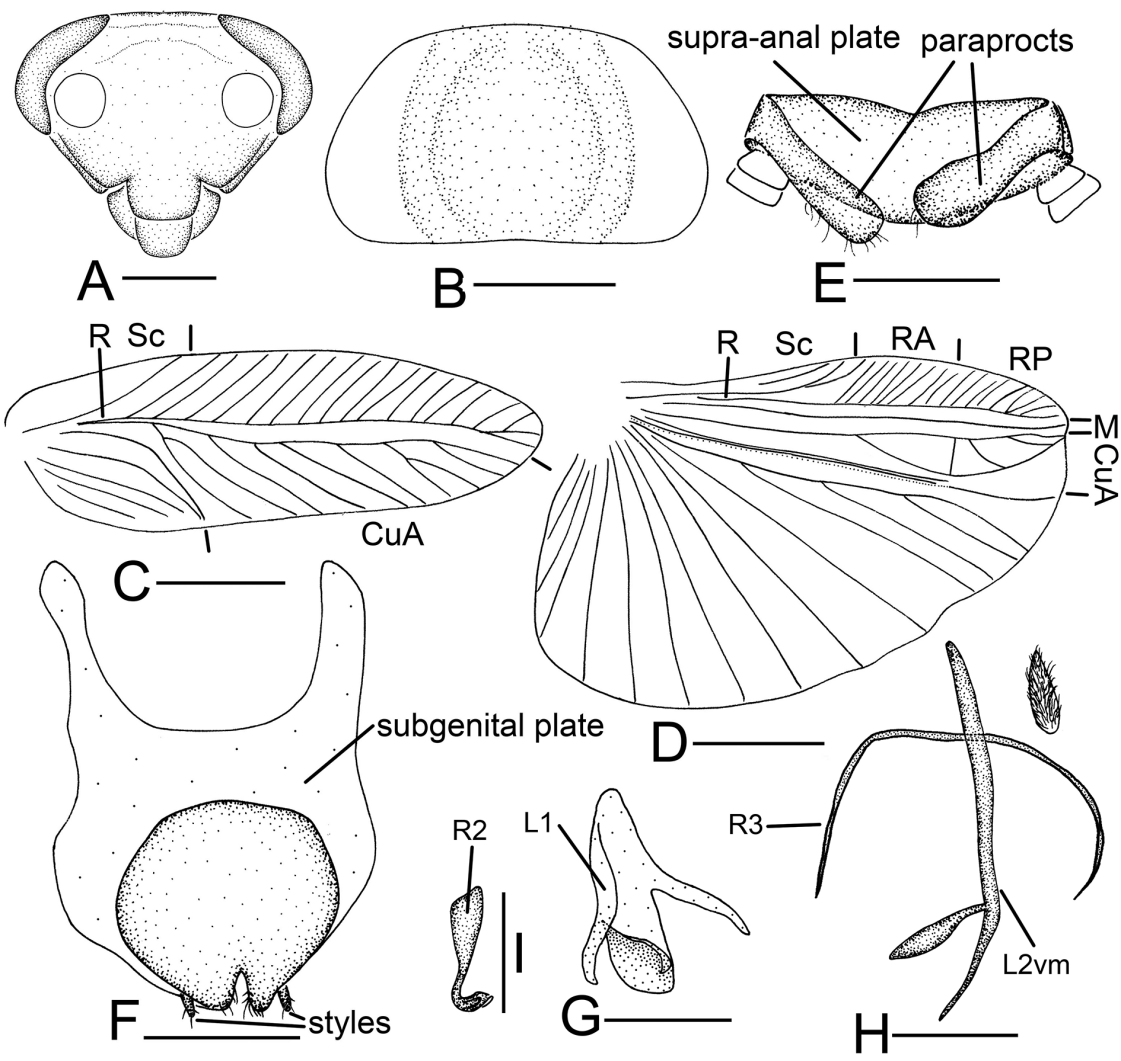
*Sorineuchora formosana* (Matsumura, 1913) male from China, Yunnan, Xishuangbanna, Mengla. **A** head, frontal view **B** pronotum **C** tegmen **D** hind wing (the dotted line indicates wing fold) **E** supra-anal plate, ventral view **F** subgenital plate, dorsal view **G** phallomere L1 **H** phallomere L2vm and R3 **I** phallomere R2. Scale bars: 0.5 mm (**A, E–I**), 1.0 mm (**B**), 2.0 mm (**C, D**).

CuA with one complete branch, between CuA and its branch existing two or three cross veins (Fig. 2D); L2vm rod-like, bifurcate; R3 shaped like a slender curved filament, lying under the L2vm; a setose membrane on the right side (Fig. 2H). Using these traits, *S. formosana* can be distinguished from its congeneric species.

**Supplement to the description provided in Roth (1998: 15–16).**

#### Measurements

(mm). Body length without cerci: male 6.8–8.3, female 7.6–8.8; overall length including tegmen: male 8.9–10.4, female 8.9–10.5; pronotum length × width: male 1.85 × 3.1, female 1.95 × 3.4; tegmen length: male 7.2–8.7, female 7.6–8.1.

**Male.** Body small, yellowish brown. Vertex slightly brown, frons yellowish white. Ocellar area yellowish white. Maxillary palpi yellowish white. Tegmina yellowish brown, veins and radial field yellowish white. Abdomen and legs yellow. Interocular space slightly narrower than distance between antennal sockets. Pronotum subelliptical, anterior and posterior margins nearly truncate.

#### Distribution.

China (Taiwan, Hainan, Yunnan).

#### Remarks.

Based on the illustrations of wings and subgenital plate in Asahina (1978, figs 8, 16, 17) and the subgenital plate and genitalia in Roth (1998, fig. 40), we identified our materials as *S. formosana*. Asahina (1978) noted that *S. formosana* allied to *S. lativitrea* from Southeast Asia. However, the differences between *S. formosana* (Fig. 11A–B) and the holotype of *S. lativitrea* (Fig. 11C–D) (size and color) make us suspect of the supposed relationship.

### Sorineuchora nigra

Taxon classificationAnimaliaBlattodeaEctobiidae

(Shiraki, 1908)

**Figure 3.**
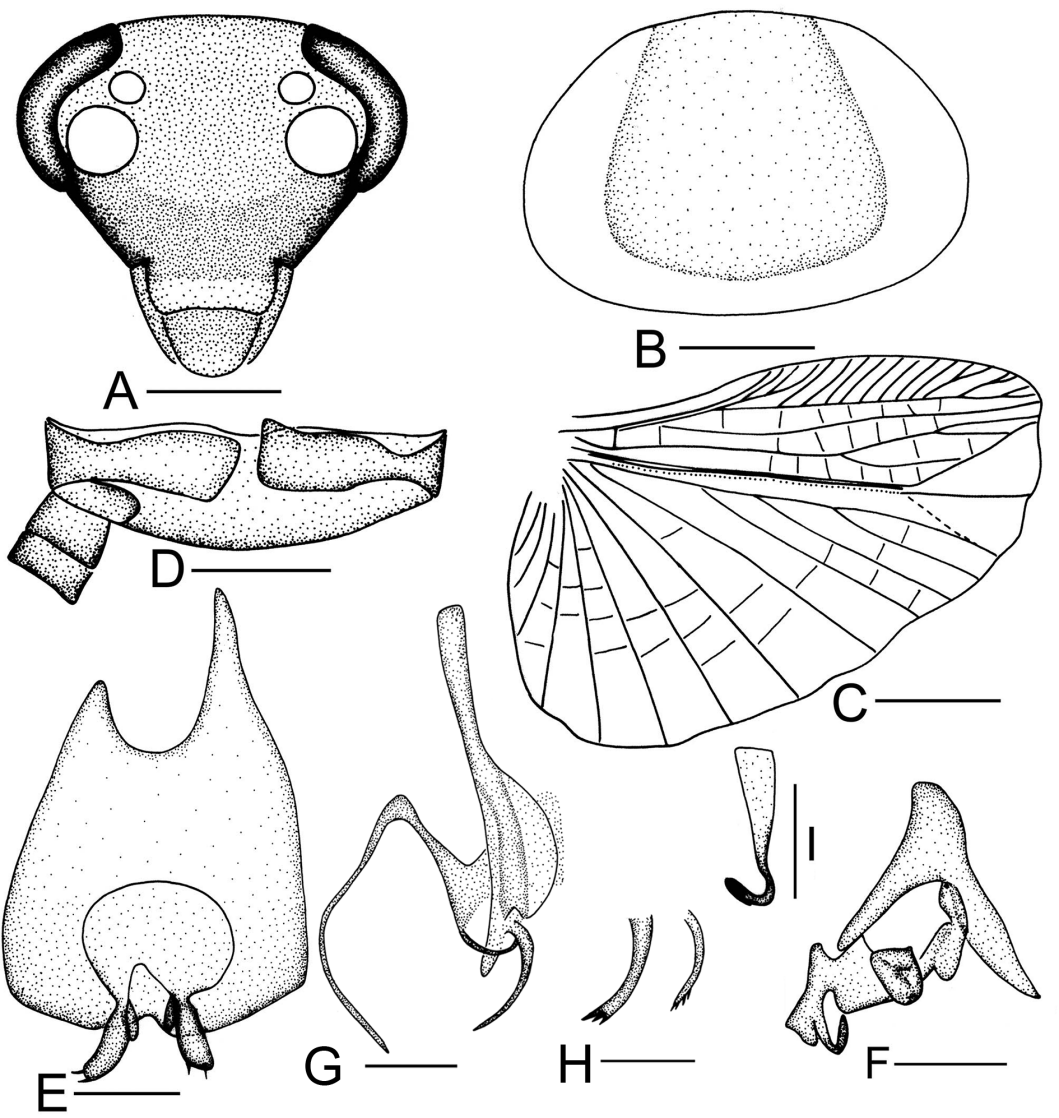
*Sorineuchora nigra* (Shiraki, 1908). **A–G, I** male from China, Chongqing, Wanzhou **H** male from China, Chongqing, Mt. Jinyunshan and China, Chongqing, Mt. Bishan Qinglong Lake **A** head, frontal view **B** pronotum **C** hind wing (the dotted line indicates wing fold) **D** supra-anal plate, ventral view **E** subgenital plate, dorsal view **F** phallomere L1 **G** phallomere L2vm and R3 **H** phallomere L2vm **I** phallomere R2. Scale bars: 0.5mm (**A, D–I**), 1.0 mm (**B**), 2.0 mm (**C**).

Figs 3 , 10B–C , 11F–G

Chorisoneura nigra Shiraki, 1908: 109 (♂); Matsumura 1913: 8; Karny 1915: 63; Hanitsch 1927: 42; Asahina 1991: 71.
Lupparia nigra (Shiraki): Shiraki 1931: 197; 1950: 59; Matsumura 1931: 1376; Asahina 1955: 204.
Balta nigra (Shiraki): Princis 1969: 978; 1971: 1143.
Sorineuchora nigra : Roth 1998: 16 (♂).
Chorisoneura setshuana Bey-Bienko, 1958: 680, 689, fig. 11 (♂♀); Liu and Zhu 2001 (synonymy).

#### Material examined

**(all deposited in IESWU). Chongqing**: 1 male, Changshou, Munanyuan, 450m, 09 June 1994, Wen-Zhu Li leg.; 1 male, Wanzhou, 1200m, 10 July 1993, Jian Yao leg.; 6 males, Fengdu, Shiping, 610m, 02–03 June 1994, You-Wei Zhang leg.; 3 males, Mt. Jinyunshan, 800m, 13 June 1994, You-Wei Zhang leg.; 6 males, Mt. Bishan, Qinglong Lake, 10 June 2006, You-Wei Zhang leg.; 1 male, Youyang, Banxi, Sandaigou, 500m, 22 May 2007, Wei-Wei Zhang leg.; 1 male, Jiangjin, Mt. Simianshan, 15 July 2007, Wei-Wei Zhang leg; 1female, Jiangjin, Mt. Simianshan, 05 June 2014, Xin-Ran Li (= Conlin McCat) leg. **Hubei**: 3 males, Mt. Dabieshan, Taohuachong, 604m, 27 June 2014, Yan Shi and Xin-Ran Li (= Conlin McCat) leg. **Sichuan**: 1 male, Huili, 2200m, 29 July 1974, collector unknown; 1 male, Mt. Emei, Qinyinge Temple, 800–1000m, 30 May 1957, You-Cai Yu leg.; 4 males, Mt. Emei, Baoguosi Temple, 550–750m, 23–24 May 1957, Fu-Xing Zhu leg. **Guangxi**: 1 male, Longzhou, Nonggang, 20 May 1985, Wei-Hua Li and Jing-Hong Zhang leg.; 1 male, Longzhou, Nonggang. 29 June 2015, light trapping, Lu Qiu and Qi-Kun Bai leg.; **Zhejiang**: 1 male, Mt. Tianmushan, 26 June 1957, Kun-Ji Yang leg. **Hunan**: 1 male, Hengyang, Mt. Hengshan, Mojingtai, 11 May 1983, Wei-Hua Li leg. **Anhui**: 1 male, Huangshan, Tangkou, Fuxi, 10 July 2014, Xin-Ran Li (= Conlin McCat) and Jian-Yue Qiu leg. **Hainan**: 1 male, Mt. Wuzhishan, 18 May 2014, Shun-Hua Gui, Xin-Ran Li (= Conlin McCat) leg. **Guizhou**: 3 males, Leishan, Mt. Leigongshan, Xiaodanjiang, 750m, 02 June 2005, Zai-Hua Yang leg.; 2 males, Tongren, Mt. Fanjingshan, 1200m, 02 June 2002, Qiong-Zhang Song leg.

#### Diagnosis.

Body is black or blackish brown without evident stripes (Fig. 11F–G); L2vm pre-apex with a curved spine-like process, the process apex with several small spines or without (Fig. 3G–H) and ventrally with R3 whose sclerite becomes filamentous and curves to the left (Fig. 3G). Using these traits, *S. nigra* can be distinguished from its congeneric species.

**Supplement to the description provided in Roth (1998: 16–17).**

#### Measurements

(mm). Body length without cerci: male 7.6–8.4, female 7.1–8.8; overall length including tegmen: male 9.6–11.0, female 9.5–9.8; pronotum length × width: male 2.05 × 3.1, female 2.0 × 3.0; tegmen length: male 7.3–8.5, female 7.1–8.2.

**Male.** Body small, black, some individuals blackish brown. Vertex black with a rudimentary dark stripe or without stripes; frons black, or vertex and upper half of frons yellowish brown, lower half brown. Pronotal disk dark brown or black, lateral and hind margins hyaline. Interocular space slightly narrower than the distance between antennal sockets. Pronotum subelliptical, anterior and posterior margins nearly truncate. Subgenital plate with a pair of stout styli, the apex slightly pointing outward. L1 consisting of several irregular seta-free sclerites (Fig. 3F); L2vm pre-apex with a curved spine-like process, the process apex with several small spines or without (Fig. 3G–H).

**Female.** Some individuals are similar to the male in color and habitus, but supra-anal plate symmetrical with hind margin rounded and subgenital symmetrical with hind margin rounded and slightly concave medially. Some individuals do vary distinctly in body color (Fig. 10C) (body brownish red). Head brownish yellow, vertex with a yellowish brown stripe. Clypeus yellowish brown. Wing veins white, legs brown, trochanter yellowish brown, abdominal brown, posterior and lateral margins milk white. We analyzed COI gene sequences of the exceptional female specimen (MF612149), and female specimen (KY349518), which is similar to male in color and male specimen (KY349519) using MEGA7 (Kumar et al. 2016), the similarity was 98.5% (MF612149 and KY349518), 99.4% (MF612149 and KY349519) and 99.1% (KY349518 and KY349519), respectively.

#### Distribution.

China (Taiwan, Chongqing, Hubei, Sichuan, Guangxi, Guizhou, Zhejiang, Hunan, Anhui, Hainan), Japan.

#### Remarks.

Roth (1998) noted that *S. nigra* and *S. setshuana* might prove to be synonyms by comparing figs 10 and 18 in Asahina (1978). Liu and Zhu (2001) synonymized *S. setshuana* and *S. undulata* with *S. nigra* without giving any details. Based on examining specimens kept in IESWU and the descriptions of *S. undulata* by Bey-Bienko (1958), there are many differences between *S. nigra* and *S. undulata* in coloration, the details of tegmina and male subgenital plate (Figs 3E, 5G, 11F–G, 11J–K). Therefore, *S. undulata* is not a synonym of *S. nigra*.

### Sorineuchora shanensis

Taxon classificationAnimaliaBlattodeaEctobiidae

(Princis, 1950)

**Figure 4.**
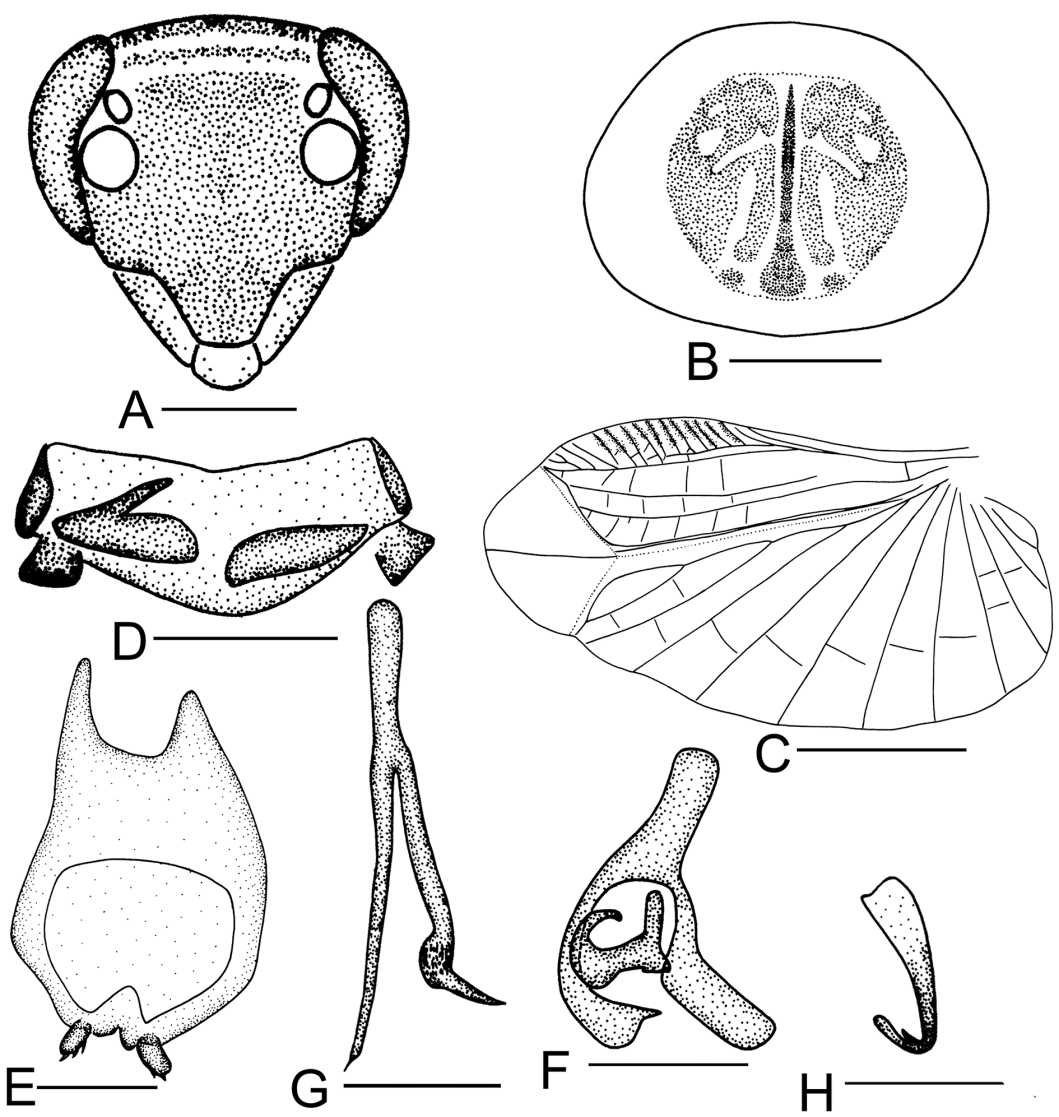
*Sorineuchora shanensis* (Princis, 1950) male from China, Yunnan, Pu’er, Simao. **A** head, frontal view **B** pronotum **C** hind wing (the dotted line indicates wing fold) **D** supra-anal plate, ventral view **E** subgenital plate, dorsal view **F** phallomere L1 **G** phallomere L2vm and R3 **H** phallomere R2. Scale bars: 0.5mm (**A, D–H**), 1.0 mm (**B**), 2.0 mm (**C**).

Figs 4 , 10D , 11H–I

Sorineuchora nigra Princis, 1950: 208, fig. 4 (♂♀).
Sorineuchora shanensis (Princis): Roth, 1998: 17, Figs 44–48 (♂♀).

#### Material examined

**(all from Yunnan, deposited in IESWU).** 1 male, Pu’er, Simao, 04 July 2004, Xiang-Rong Xu leg.; 2 females 2 males, Xishuangbanna, Mengyang, 800m, 06 June 1991, Ying-Lun Wang and Run-Gang Tian leg.; 3 males, Pu’er, Simao, Meizihu, 19 July 2009, Zong-Qing Wang leg.; 1 male, Pu’er, Simao, Meizihu, 22 May 2016, Lu Qiu, Zhi-Wei Qiu leg.; 2 females 3 males, Lincang, Nansan, 1010m, 08 July 2007, Li-Jun Cai leg.; 1 female 1 male, Menglun, 30–31 July 2009, Zong-Qing Wang leg.; 1 female, Pu’er, Xiaoheijiang, 24 July 2009, Zong-Qing Wang leg.; 2 females 1 male, Pu’er, Yixiangzhen, Cilincun, 02 May 2013, Zong-Qing Wang leg.

#### Diagnosis.

Vertex dark with a pair of white or yellow transverse stripes; pronotal disk black, with symmetrical white or yellow markings (Figs 4B, 10D, 11H); L2vm with one branch (Fig. 4G); tegmen dark, veins white or dark (Figs 10D, 11H) Using these traits, *S. shanensis* can be distinguished from its congeneric species.

**Supplement to the description provided in Roth (1998: 17–19).**

#### Measurements

(mm). Body length without cerci: male 4.9–5.4, 5.8–6.5; overall length including tegmen: male 7.5–8.5, female 7.0–8.5; pronotum length × width: male 1.85 × 2.6, female 1.75 × 2.55; tegmen length: male 5.4–6.0, female 5.2–6.5.

**Male.** Body small, dark. Vertex dark with a pair of white or yellow transverse stripes. Clypeus reddish brown. Antennae with first six basal antennomeres black, the rest brown. Pronotal disk black, with symmetrical white or yellow markings (Figs 4B, 10D, 11H), lateral margins hyaline. Tegmen dark, veins white or dark (Figs 10D, 11H). Pronotum subelliptical, posterior margin nearly truncate. Supra-anal plate with hind margin convex, or some individuals with hind margin weakly concave, paraprocts slightly dissimilar and sheet-like (Fig. 4D). The styli with small spines at preapical and inner sides (Fig. 4E). L1 consisting of several irregular seta-free sclerites (Fig. 4F).

#### Distribution.

China (Yunnan); Myanmar.

#### Remarks.

According to the stripes on the vertex (Fig. 11I), the markings on the pronotal disk (Figs 4B, 11H) and the color of vein of the tegmen (Figs 10D, 11H), this species is easily recognized.

### Sorineuchora undulata

Taxon classificationAnimaliaBlattodeaEctobiidae

(Bey-Bienko, 1958)

Figs 5 , 11J–K

Chorisoneura undulata Bey-Bienko, 1958: 680, 689 (♂).
Sorineuchora undulata (Bey-Bienko): Roth 1998: 21 (♂).

#### Material examined

**(all deposited in IESUW).** 1 male, China, Yunnan, Xishuangbanna, Wangtianshu, 23 May 2016, Zhi-Wei Qiu and Lu Qiu leg.

#### Diagnosis.

**Figure 5.**
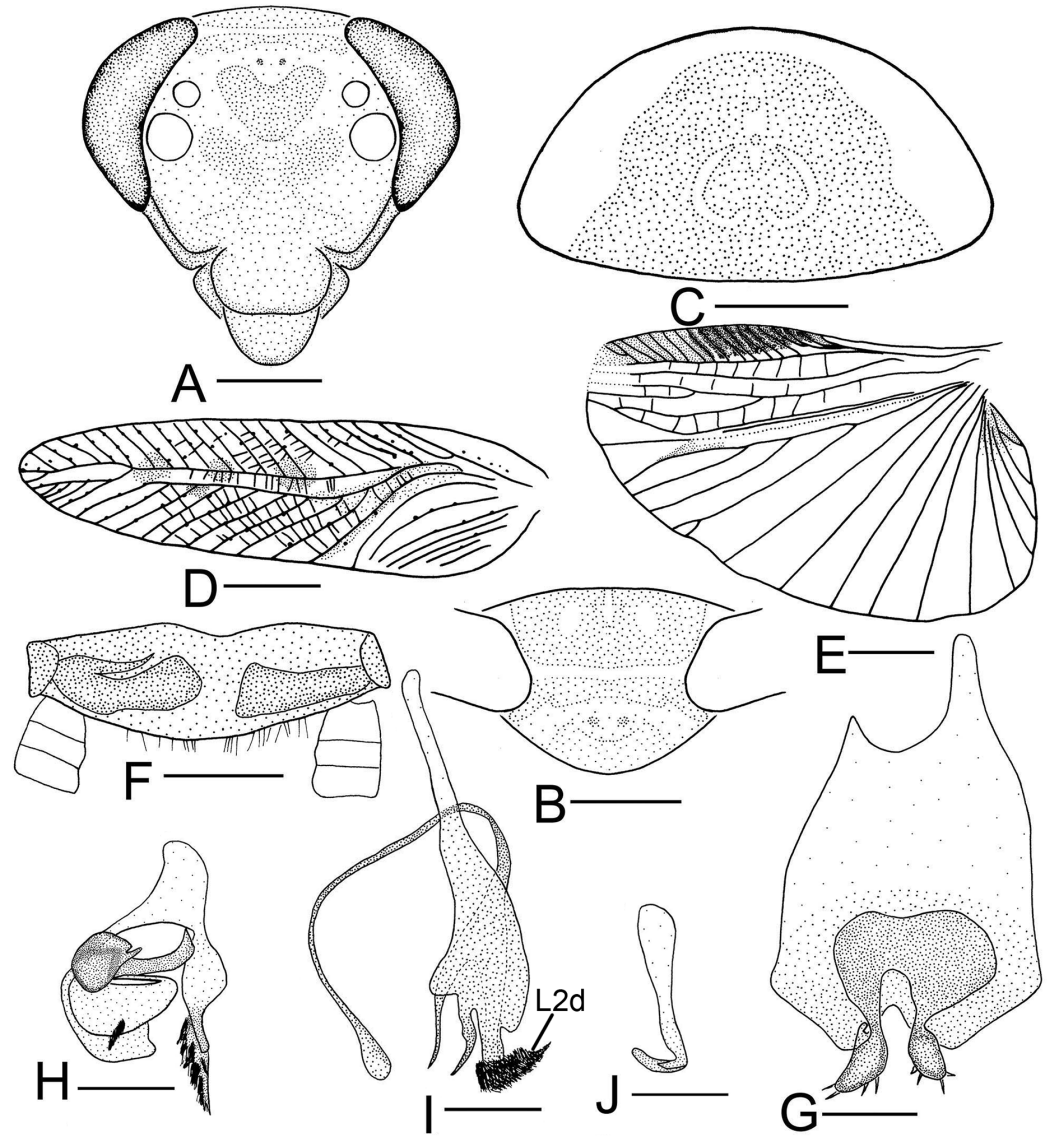
*Sorineuchora undulata* (Bey-Bienko, 1958) male from China, Yunnan, Xishuangbanna, Wangtianshu. **A** head, frontal view **B** vertex **C** pronotum **D** tegmen **E** hind wing (the dotted line indicates wing fold) **F** supra-anal plate, ventral view **G** subgenital plate, dorsal view **H** phallomere L1 **I** phallomere L2vm and R3 **J** phallomere R2. Scale bars: 0.5 mm (**A, B, F–J**), 1.0 mm (**C**), 2.0 mm (**D, E**).

On the frons between the ocelli with the V shaped blotch (Fig. 5A); tegmina yellowish brown with four dark spots on the radius and many black dots on veins (Fig. 5D); L1 with setae on the right apex; L2vm with its middle inflated, the apex with two branches, L2d setose, R3 right pre-apex lying under the L2vm (Fig. 5I). Using these traits, *S. undulata* can be distinguished from its congeneric species.

**Supplement to the description provided in Roth (1998).**

#### Measurements

(mm). Body length without cerci: 8.9; overall length including tegmen: male 10.8; pronotum length × width: male 2.2 × 4.1; tegmen length: male 9.0.

**Male.** Tegmina yellowish brown with four dark spots on the radius and many black dots on veins (Figs 5D, 11J). Interocular space slightly narrower than the distance between antennal sockets. Paraprocts sheet-like and the left with a branch (Fig. 5F). L1 consisting of several irregular sclerites, the right apex with setae (Fig. 5H); L2vm with its middle inflated, the apex with two branches, L2d setose, R3 right pre-apex lying under the L2vm (Fig. 5I); hooked phallomere (R2) on the right side, with a preapical incision.

#### Distribution.

China (Yunnan).

#### Remarks.

The dots on tegmina of *S. undulata* resemble that of *S. punctipennis*, it differs in having longer body, shorter tegmina, and a strong wavy and bent CuA of the hind wing (Fig. 5E).

### Sorineuchora bivitta

Taxon classificationAnimaliaBlattodeaEctobiidae

(Bey-Bienko, 1969)

Figs 6 , 12A–B

Chorisoneura bivitta Bey-Bienko, 1969: 838, fig. 17 (♂).
Sorineuchora bivitta : Roth 1998: 20 (♂).

#### Material examined.

**Deposited in IESWU**: 1 male, Yunnan, Hekou, Nanxi, Huayudong Forest Park, 20–21 April 2009, Wei-Wei Zhang leg.; 2 males, Guizhou, Maolan, Yongkang, 25–28 May 1998, Qiong-Zhang Song leg.; 1 male, Guizhou, Wangmo, 06 June 1982, Ping-Zhang Feng leg.; 1 male, Fujian, Sanming, Shaxian, 23 May 1977, Qing-Dong Luo leg.; 1 male, Guangxi, Longzhou, 31 May 1997, Mao-Fa Yang leg.; 1 male, Guangxi, Chongzuo, Banli National Nature Reserve, 174m, 31 May 2009, Wei-Wei Zhang leg.; 1 male, Guangxi, Hechi, Mt. Daqingshan, 14 May 1963, Si-Kong Liu leg.; 1 male, Hainan, 25 May 1997, Mao-Fa Yang leg. **Deposited in MHBU**: 1 male, China, Guangdong, Huizhou, Mt. Nankunshan, 25 July 2010, Hao-Yu Liu leg.

**Figure 6.**
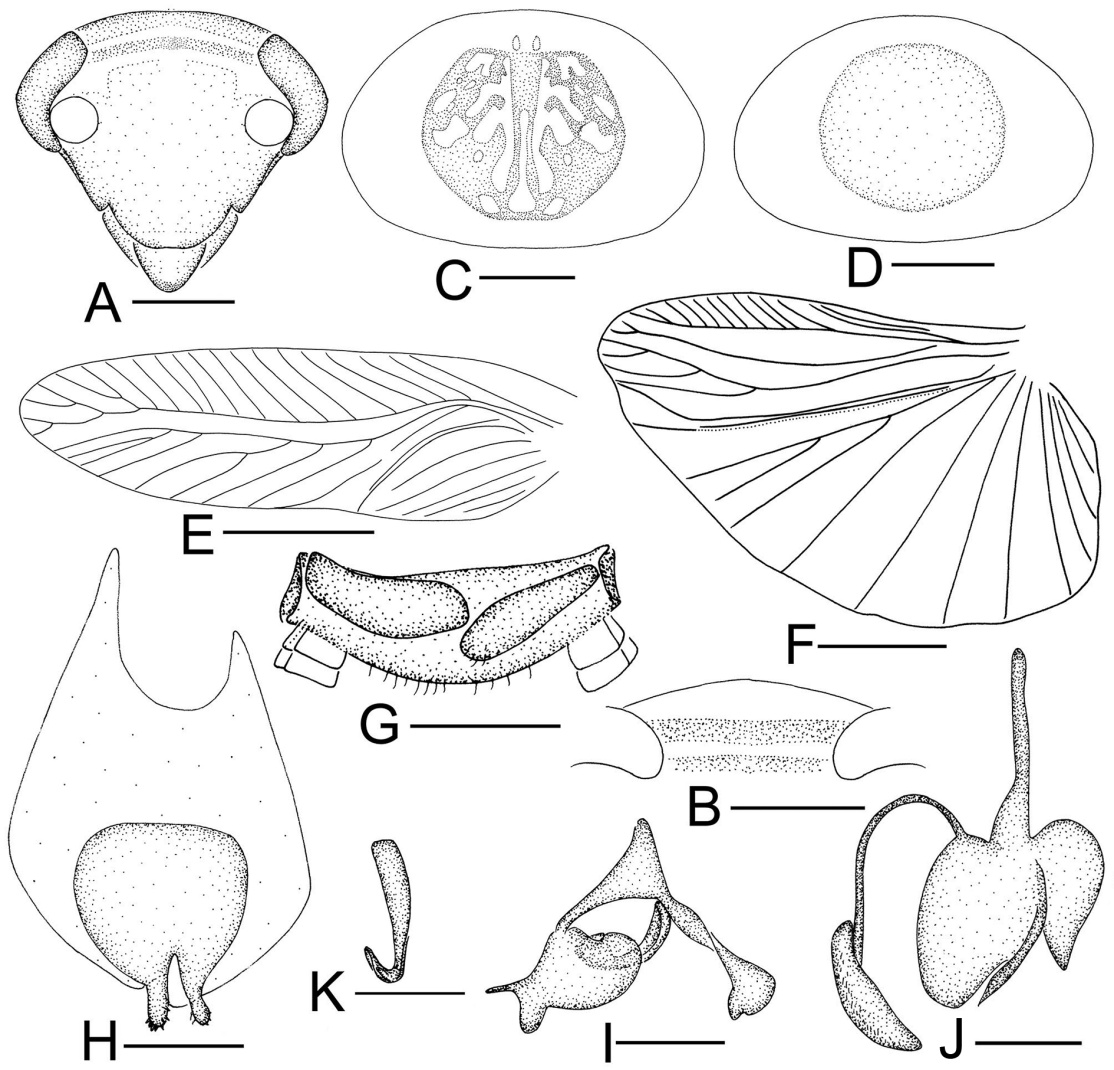
*Sorineuchora bivitta* (Bey-Bienko, 1969). **A–C, E–K** male from China, Yunnan, Hekou, Nanxi, Huayudong Forest Park **D** male from China, Guangxi, Longzhou **A** head, frontal view **B** vertex **C–D** pronotum **E** tegmen **F** hind wing (the dotted line indicates wing fold) **G** supra-anal plate, ventral view **H** subgenital plate, dorsal view **I** phallomere L1 **J** phallomere L2vm and R3 **K** phallomere R2. Scale bars: 0.5mm (**A–B, G–K**), 1.0 mm (**C, D**), 2.0 mm (**E, F**).

#### Diagnosis.

Vertex with two black stripes, the regions between them yellow (Figs 6B, 12B); L2vm with inflated apex and the left with filamentous sclerite whose apex inflated (Fig. 6J). Using these traits, *S. bivitta* can be distinguished from its congeneric species.

**Supplement to the description provided in Roth (1998: 20–21).**

#### Measurements

(mm). Body length without cerci: male 6.5–7.9, female 6.5–7.8; overall length including tegmen: male 9.3–10.5, female 9.5–11.0; pronotum length × width: male 1.95 × 3.05, female 1.95 × 2.95; tegmen length: male 7.9–9.0, female 8.2–9.0.

**Male.** In some individuals, the coloration of the pronotal disk is blackish brown without stripes (Fig. 6D), and in other individuals, the pronotal disk has a circular dark brownish spot and dense markings consisting of black spots and longitudinal and oblique stripes (Fig. 6C). Abdomen dark red-brown. Legs black-brown. Cerci apex yellowish brown. Interocular space as wide as or narrower than the distance between antennal sockets. Fifth segment of maxillary palpus longer than the fourth. Pronotum subelliptical, anterior and posterior margins nearly truncate. Tegmina and wings fully developed extending beyond end of abdomen, the former with oblique CuA. Hind-wing radial field narrow, R with oblique branches of which some apical ones bifurcated, M bent, without branches or with a small branch at the apex, CuA with three complete branches. Front femur Type C_2_, pulvilli on four proximal tarsomeres, tarsal claws asymmetrical, arolia present. Abdominal terga unspecialized. L1 consisting of several irregular seta-free sclerites (Fig. 6I); L2vm with inflated apex and the left with filamentous sclerite whose apex inflated (Fig. 6J); hooked phallomere (R2) on the right side with a preapical incision.

#### Distribution.

China (Yunnan, Guizhou, Fujian, Guangxi, Hainan, Guangdong).

#### Remarks.

**Figure 7.**
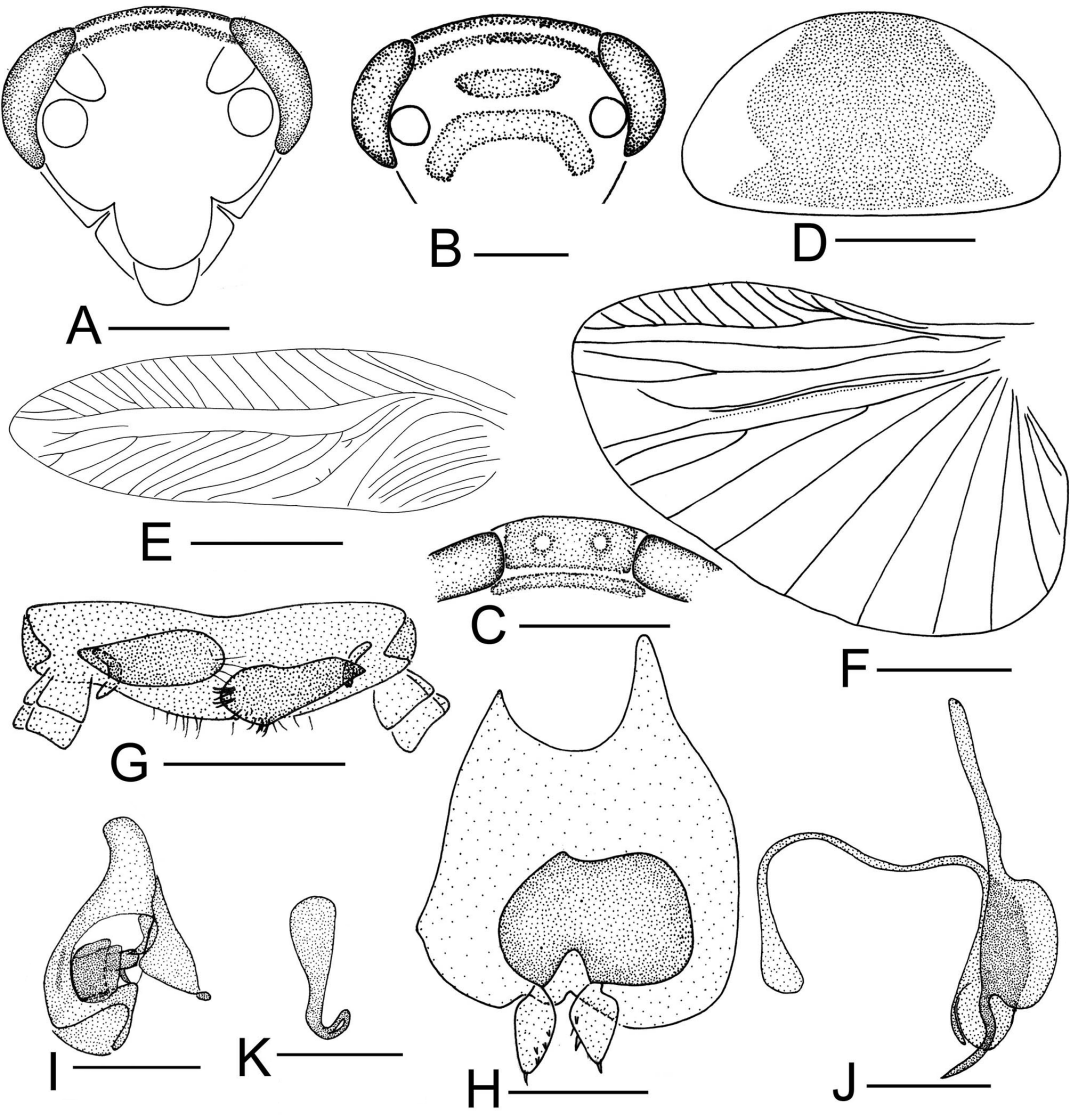
*Sorineuchora bimaculata* sp. n. **A** Paratype, male from China, Chongqing, Wulong, Wanfeng **B–K** Holotype **A–B** heads, frontal view **C** vertex **D** pronotum **E** tegmen **F** hind wing (the dotted line indicates wing fold) **G** supra-anal plate, ventral view **H** subgenital plate, dorsal view **I** phallomere L1 **J** phallomere L2vm and R3 **K** phallomere R2. Scale bars: 0.5 mm (**A–C, G–K**), 1.0 mm (**D**), 2.0 mm (**E, F**).

The color of *S. bivitta* resembles that of *S. bimaculata* sp. n. (Fig. 12A–D), but the former is easily distinguished from the latter by the markings on the vertex (Figs 6B, 7C) and the shape of styli (Figs 6H, 7H).

### Sorineuchora bimaculata sp. n.

Taxon classificationAnimaliaBlattodeaEctobiidae

http://zoobank.org/65DEB5F4-2CCC-4042-B5D0-6DBDD6C192B7

Figs 7 , 12C–D

#### Type material.

**Holotype** male (IESWU), China, Guizhou, Luodian, June 1981, unknown leg. **Paratypes** (deposited in IESWU). 1 male, Guizhou, Maolan, Xiaoqikong, 30 May 1998, Qiong-Zhang Song leg.; 1 male, Yunnan, Xishuangbanna, Meng’a, 1050–1080m, 20 June 1958, Shu-Yong Wang leg.; 1 male, Chongqing, Wulong, Wanfeng, 800m, 7 July 1989, Long-Long Yang leg.; 1 male, Hubei, Luotian, Mt. Dabieshan, 01–02 July 2014, Yan Shi and Xin-Ran Li (= Conlin McCat) leg.

#### Diagnosis.

Upper half of vertex brown, with two round yellowish brown spots in the middle (Fig. 7C); a pair of styli have three to six small spines at the apex and inner margins; L2vm the middle inflated, pre-apex curved and apex acute, R3 arched and filament, the apex inflated (Fig. 7J). Using these traits, the new species can be distinguished from its congeneric species.

#### Description.

Measurements (mm). **Holotype**, body length without cerci: 7.6, overall length including tegmen: 8.8; pronotum length × width: 1.9 × 2.7; tegmen length: 7.4. **Paratypes**, body length without cerci: 7.0–7.8; overall length including tegmen: 9.0–11.0; pronotum length × width: 1.75 × 2.8; tegmen length: 8.0–9.0.

**Male.** Body small, dark brown. Upper half of vertex brown, with two round yellowish brown spots in the middle (Fig. 7C), lower half reddish brown, with a black transverse stripe. Frons brown to yellowish brown and without a stripe (Fig. 7A), or with a bent light brown stripe (Fig. 7B). Pronotum yellowish brown without stripes, or brown with a longitudinal light brown stripe, lateral margins hyaline. Tegmen reddish brown. Abdomen brown, lateral and hind margins light. Legs yellowish brown, the coxa brown.

Interocular space as wide as, or wider than, the distance between ocelli, and narrower than the distance between antennal sockets. Fifth segment of maxillary palpus longer than the fourth. Pronotum subelliptical, posterior margin truncate. Tegmina and wings fully developed extending beyond end of abdomen, the former with oblique CuA. Hind-wing R with oblique branches, M without branch, CuA with one branch, apical triangle evident. Front femur Type C_2_, pulvilli on four proximal tarsomeres, tarsal claws asymmetrical, arolia present. Abdominal terga unspecialized.

Supra-anal plate short and symmetrical, paraprocts similar and sheet-like (Fig. 7G). Subgenital plate with subsymmetrical hind margin, a pair of styli which have three to six small spines at the apex and inner margins, situated almost in the middle of hind margin, the interstylar margin slightly concave (Fig. 7H). L1 consisting of several irregular seta-free sclerites (Fig. 7I); middle of L2vm inflated, pre-apex curved and apex acute; R3 arched and filament, the apex inflated (Fig. 7J); hooked phallomere (R2) on the right side, with a preapical incision.

**Female.** Unknown.

#### Distribution.

China (Guizhou; Yunnan; Hubei; Chongqing).

#### Etymology.

Latin word *bimaculata* refers to the two round yellowish brown spots on vertex.

#### Remarks.

See remarks under the *S. bivitta*.

### Sorineuchora viridis sp. n.

Taxon classificationAnimaliaBlattodeaEctobiidae

http://zoobank.org/107BBC3B-8716-4737-8F45-CCAB558F4FD3

**Figure 8.**
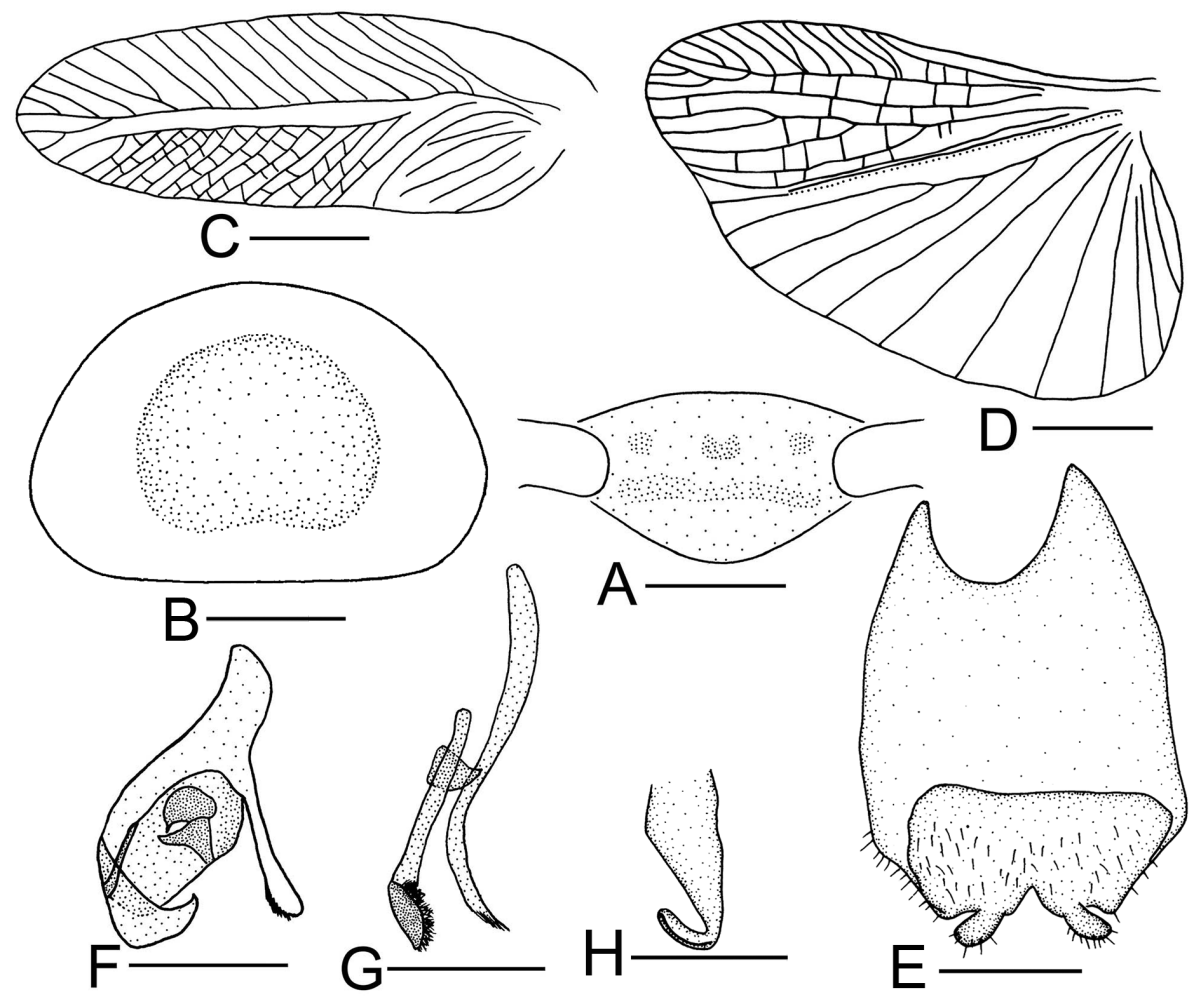
*Sorineuchora viridis* sp. n. holotype. **A** vertex **B** pronotum **C** tegmen **D** hind wing (the dotted line indicates wing fold) **E** subgenital plate, dorsal view **F** phallomere L1 **G** phallomere L2vm and R3 **H** phallomere R2. Scale bars: 0.5 mm (**A, E–H**), 1.0 mm (**B**), 2.0 mm (**C, D**).

Figs 8 , 10A , 12E–F

#### Type material.

**Holotype** male (IESWU), China, Hainan, Mt. Bawangling. 13 April 2016, light trapping, Jian-Yue Qiu leg. **Paratypes** (all from Hainan, deposited in MHBU). 1 male, Mt. Bawangling, 11–12 May 2007, Yi-Bin Ba and Jun-Tong Lang leg.; 3 males, Baisha, Nankai, 450m, 25–26 June 2008, Yi-Bin Ba and Jun-Tong Lang leg.

#### Diagnosis.

The color of the insects is green when they are alive (Fig. 10A), but it will become pale green or pale yellow when dried or kept in alcohol (Fig. 12E–F); vertex with three dark spots and a dark transverse stripe (Fig. 8A); Tegmina with white dots on the veins (Figs 10A, 12E); appendicular field almost disappearing (Fig. 8D); L1 with black setae on the right apex (Fig. 8F); L2vm rod-like, connected with R3 by sclerite (Fig. 8G). Using these traits, the new species can be distinguished from its congeneric species.

#### Description.

Measurements (mm). **Holotype**, body length without cerci: 7.1; overall length including tegmen: 9.8; pronotum length × width: 2.0 × 3.1; tegmen length, 8.5. **Paratypes**, body length without cerci: 6.7–7.7; overall length including tegmen: 9.4–11.2; pronotum length × width: 2.35 × 3.05; tegmen length, 8.0–9.0.

**Male.** Body small, light green when alive (Fig. 10A), but it will turn pale yellow or pale green when dried or kept in alcohol (Fig. 12E–F). The morphological description here is with the specimen dried.

Vertex with three dark spots, on the frons between the ocelli with a narrow dark transverse tripe (Fig. 8A). Maxillary palpi yellowish white, antennae yellow. Pronotum hyaline. Tegmina and wings hyaline. The former’s veins light with scattered white dots (Fig. 12E–F). Abdomen and legs yellowish white.

Interocular space as wide as or slightly narrower than the space between antennal sockets. Fifth segment of maxillary palpus longer than the fourth. Pronotum subelliptical, anterior and posterior margins nearly truncate. Tegmina and wings fully developed extending beyond end of abdomen, the former with oblique CuA. Hind-wing R with oblique branches, M without branches, CuA with three branches, appendicular field almost disappearing. Front femur Type C_2_, pulvilli on four proximal tarsomeres, tarsal claws asymmetrical, arolia present. Abdominal terga unspecialized.

Supra-anal plate with hind margin rounded, paraprocts simple. Subgenital plate with subsymmetrical hind margin, a pair of styli with small setae, hind margin medially deflexed forming a short, longitudinal keel-like ridge, interstylar margin almost straight when flattened (Fig. 8E). L1 consisting of several irregular sclerites, the right apex with black setae (Fig. 8F); L2vm rod-like, connected with R3 whose apex has many setae by a sclerite (Fig. 8G); hooked phallomere (R2) on the right side, with a preapical incision.

**Female.** Unknown

#### Distribution.

China (Hainan).

#### Etymology.

Latin word *viridis*, meaning green, refers to the color of this species when alive.

#### Remarks.

*Sorineuchora viridis* sp. n. is similar to *S. javanica* (Caudell, 1927) in color (when faded) and subobsolete apical triangle. But *S. viridis* sp. n. differs from *S. javanica* in details of vertex, dots on the tegmina, and median and left phallomeres.

### Sorineuchora hispida sp. n.

Taxon classificationAnimaliaBlattodeaEctobiidae

http://zoobank.org/A8A46CC5-E282-4835-BA18-F0773A672219

Figs 9 , 12G–J

#### Type material.

**Holotype** male (IESWU), China, Guangxi, Guiping, Longtan Park, 30 May–02 June 2014, light trapping, Shun-Hua Gui leg. **Paratypes.** 1 female, 3 males, same data as holotype.

#### Diagnosis.

**Figure 9.**
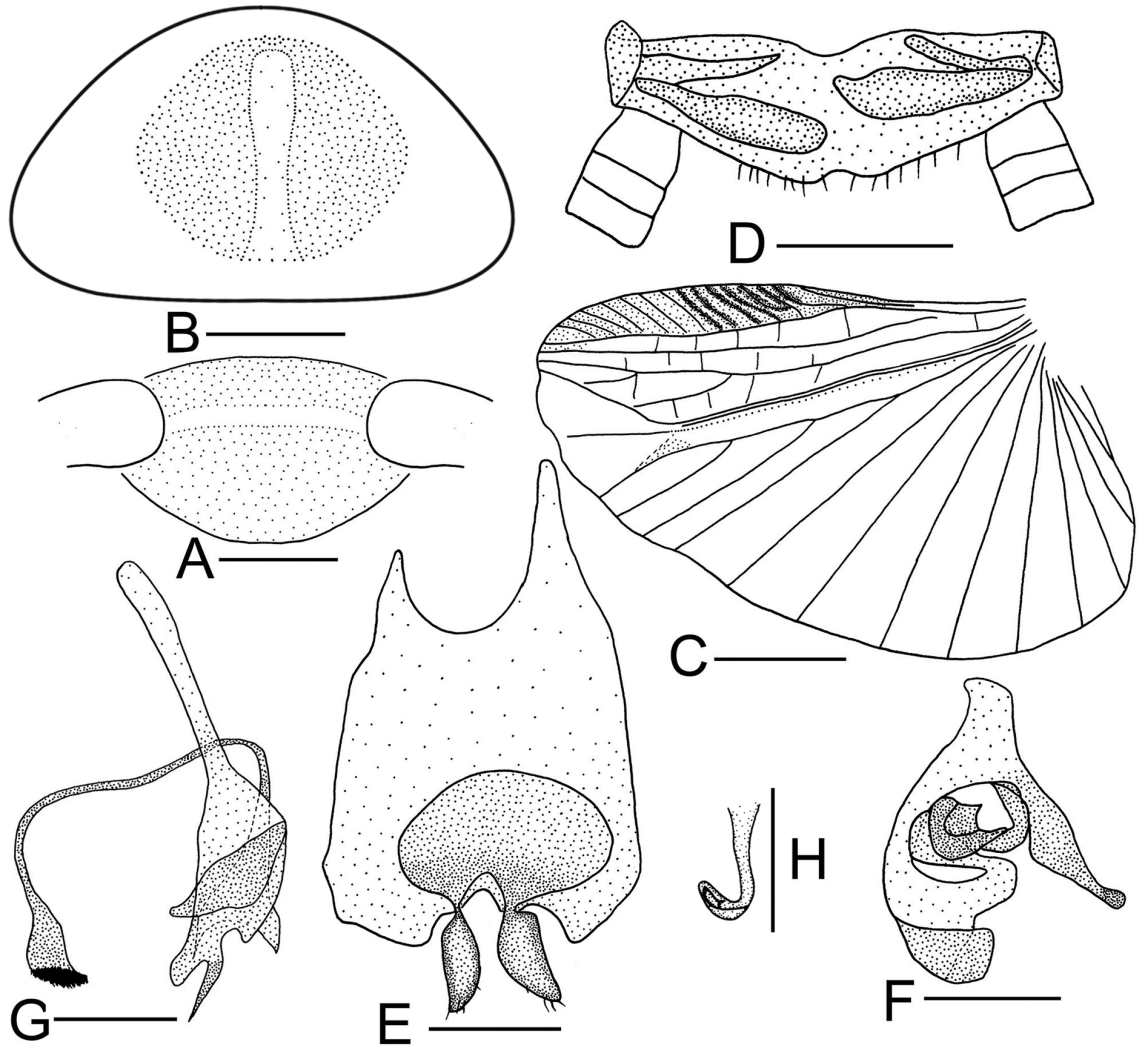
*Sorineuchora hispida* sp. n. holotype. **A** vertex **B** pronotum **C** hind wing (the dotted line indicates wing fold) **D** supra-anal plate, ventral view **E** subgenital plate, dorsal view **F** phallomere L1 **G** phallomere L2vm and R3 **H** phallomere R2. Scale bars: 0.5 mm (**A, D–H**), 1.0 mm (**B**), 2.0 mm (**C**).

Pronotal disk brown, with a yellowish brown, longitudinal stripe (Figs 9B, 12G); paraprocts similar, sheet-like, with a branch (Fig. 9D); the left apex of R3 with many setae (Fig. 9G). Using these traits, the new species can be distinguished from its congeneric species.

#### Description.

Measurements (mm). **Holotype**, male, body length without cerci: 7.0; overall length including tegmen: 8.6; pronotum length × width: 1.7 × 2.7; tegmen length: 7.5. **Paratypes**, body length without cerci: male 6.4–7.6, female 6.5; overall length including tegmen: male 8.8–9.2, female 9.1; pronotum length × width, male 2.05 × 2.75, female 1.7 × 2.8; tegmen length, male 7.5–7.6, female 7.8.

**Male.** Body small, brown. Lower half vertex yellowish brown, with one white transverse stripe (Fig. 9A). Antennae with first three basal antennomeres light yellow, the rest brown. Pronotal disk brown, with a yellowish brown, longitudinal stripe (Figs 9B, 12G), lateral margins hyaline. Hind-wing radial field brown. Legs brownish yellow. Abdomen black brown, the hind margins light.

Interocular space as wide as the distance between antennal sockets. Fifth segment of maxillary palpus longer than the fourth. Pronotum subelliptical, posterior margin truncate. Tegmina and wings fully developed, extending beyond end of abdomen. Hind-wing RA and RP parallel and inflated, M without branches, CuA with two branches, apical triangle evident. Front femur Type C_2_, pulvilli on four proximal tarsomeres, tarsal claws asymmetrical, arolia present. Abdominal terga unspecialized.

Supra-anal plate with hind margin rounded and weakly concave medially, lateral margins oblique, paraprocts similar, sheet-like, with a branch respectively (Fig. 9D). Subgenital plate with subsymmetrical hind margin, a pair of styli similar, both apexes with several asymmetrically distributed spines (Fig. 9E). L1 consisting of several irregular seta-free sclerites (Fig. 9F); L2vm with the middle inflated, apex thin and acute, the left apex of R3 with many seta (Fig. 9G); hooked phallomere (R2) on the right side, with a preapical incision.

**Female.** Similar to the male, but the pronotum with longitudinal and oblique markings, and subgenital plate with hind margin truncate.

#### Distribution.

China (Guangxi).

#### Etymology.

Latin word *hispida* means rough, shaggy, hairy, referring to the left apex of R3 with many setae.

**Figure 10.**
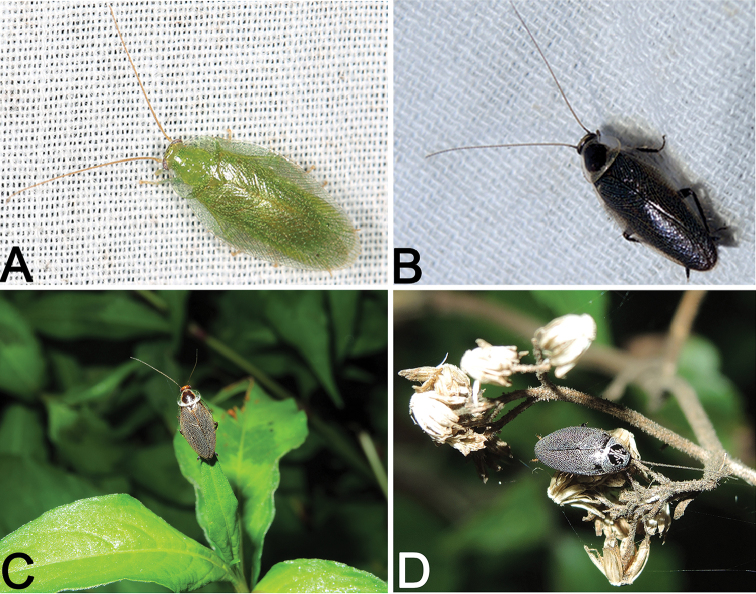
*Sorineuchora viridis* sp. n., *Sorineuchora nigra* and *Sorineuchora shanensis* in the wild. **A** *S. viridis* sp. n. holotype, photographed by Ling-Xiao Chang **B** *S. nigra* male from China, Guangxi, Longzhou, Nonggang. 29 June 2015, light trapping, photographed by Lu Qiu **C** *S. nigra* female from China, Chongqing, Jiangjin, Mt. Simianshan, 05 June 2014, photographed by Xin-Ran Li (= Conlin McCat) **D** *S. shanensis* male from China, Yunnan, Pu’er, Simao, Meizihu, 22 May 2015, photographed by Lu Qiu.

**Figure 11.**
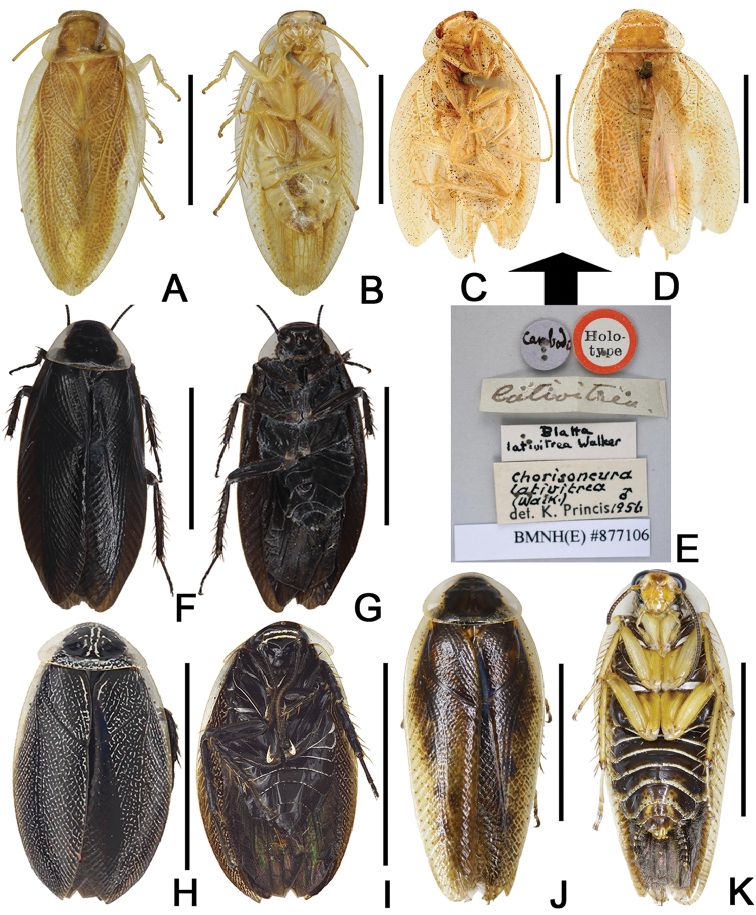
Habitus. **A, B** *S. formosana* (Matsumura, 1913) male from China, Hainan, Ledong, Mt. Jianfengling, 1050m, dorsal and ventral views **C, D, E** (labels) *S. lativitrea* (Walker, 1868) (to compare with *S. formosana*) holotype (copyright Natural History Museum, London), dorsal and ventral views **F, G** *S. nigra* (Shiraki, 1908) male from China, Hubei, Mt. Dabieshan, Taohuachong, dorsal and ventral views **H, I** *S. shanensis* (Princis, 1950) male from China, Yunnan, Lincang, Nansan, dorsal and ventral views **J, K** *S. undulata* (Bey-Bienko, 1958) male from China, Yunnan, Xishuangbanna, Wangtianshu, dorsal and ventral view. Scale bars: 5 mm.

**Figure 12.**
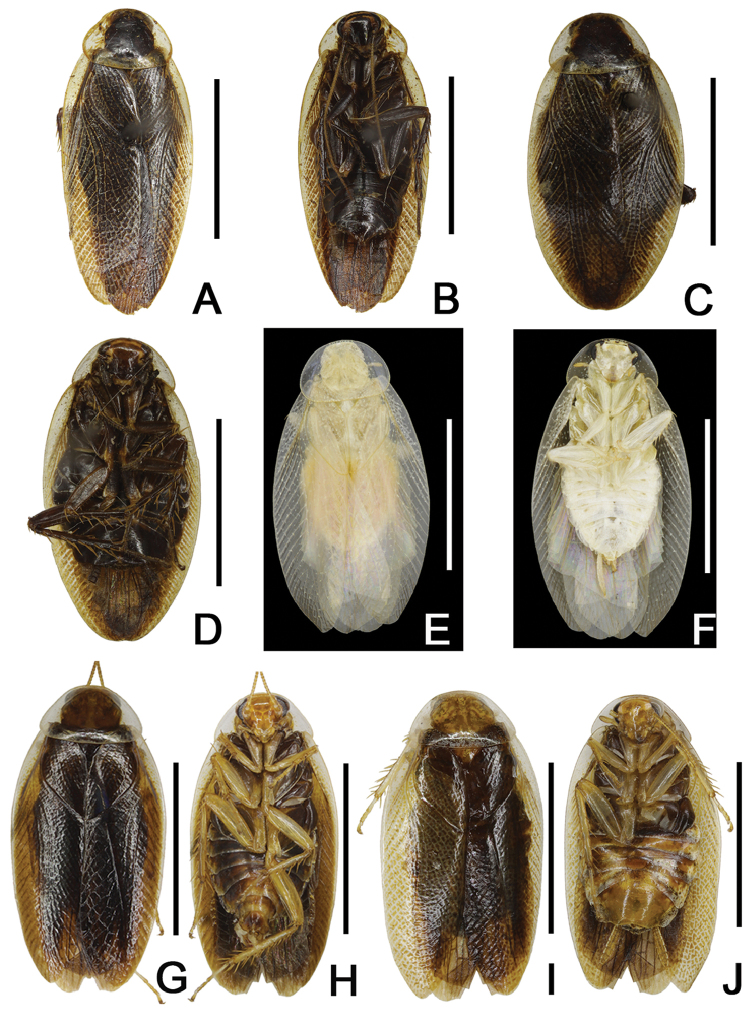
Habitus. **A, B** *S. bivitta* (Bey-Bienko, 1969) male from China, Guangxi, Hechi, Mt. Daqingshan, dorsal and ventral views **C, D** *S. bimaculata* sp. n. paratypes, male from China, Chongqing, Wulong, Wanfeng, dorsal and ventral views **E, F** *S. viridis* sp. n. holotype, dorsal and ventral views **G–J** *S. hispida* sp. n. **G–H** male paratypes, dorsal and ventral views **I, J** female paratypes, dorsal and ventral view. Scale bars: 5 mm.

## Supplementary Material

XML Treatment for Sorineuchora

XML Treatment for Sorineuchora formosana

XML Treatment for Sorineuchora nigra

XML Treatment for Sorineuchora shanensis

XML Treatment for Sorineuchora undulata

XML Treatment for Sorineuchora bivitta

XML Treatment for Sorineuchora bimaculata

XML Treatment for Sorineuchora viridis

XML Treatment for Sorineuchora hispida
